# Hydrothermal Microflow Technology as a Research Tool for Origin-of-Life Studies in Extreme Earth Environments

**DOI:** 10.3390/life7040037

**Published:** 2017-10-02

**Authors:** Kunio Kawamura

**Affiliations:** Department of Human Environmental Studies, Hiroshima Shudo University, Ozuka-higashi, Asaminami-ku, Hiroshima 731-3195, Japan; kawamura@shudo-u.ac.jp; Tel.: +81-82-830-1946

**Keywords:** hydrothermal, reaction kinetics, in situ spectroscopy, millisecond time scale, RNA, protein, mineral, high temperature and pressure, Hadean environment

## Abstract

Although studies about the origin of life are a frontier in science and a number of effective approaches have been developed, drawbacks still exist. Examples include: (1) simulation of chemical evolution experiments (which were demonstrated for the first time by Stanley Miller); (2) approaches tracing back the most primitive life-like systems (on the basis of investigations of present organisms); and (3) constructive approaches for making life-like systems (on the basis of molecular biology), such as in vitro construction of the RNA world. Naturally, simulation experiments of chemical evolution under plausible ancient Earth environments have been recognized as a potentially fruitful approach. Nevertheless, simulation experiments seem not to be sufficient for identifying the scenario from molecules to life. This is because primitive Earth environments are still not clearly defined and a number of possibilities should be taken into account. In addition, such environments frequently comprise extreme conditions when compared to the environments of present organisms. Therefore, we need to realize the importance of accurate and convenient experimental approaches that use practical research tools, which are resistant to high temperature and pressure, to facilitate chemical evolution studies. This review summarizes improvements made in such experimental approaches over the last two decades, focusing primarily on our hydrothermal microflow reactor technology. Microflow reactor systems are a powerful tool for performing simulation experiments in diverse simulated hydrothermal Earth conditions in order to measure the kinetics of formation and degradation and the interactions of biopolymers.

## 1. Introduction

A number of investigations regarding the origin of life have been carried out based on experiments that simulate primitive Earth conditions in order to determine the main prebiotic materials and reactions that contributed to the formation of primitive life-like systems. Our knowledge regarding primitive Earth environments has gradually improved through continuous efforts in geological chemistry, planetary science, and paleontology. As a result, simulation experiments are capable of being adapted for plausible Earth environments. 

**Figure 1:**
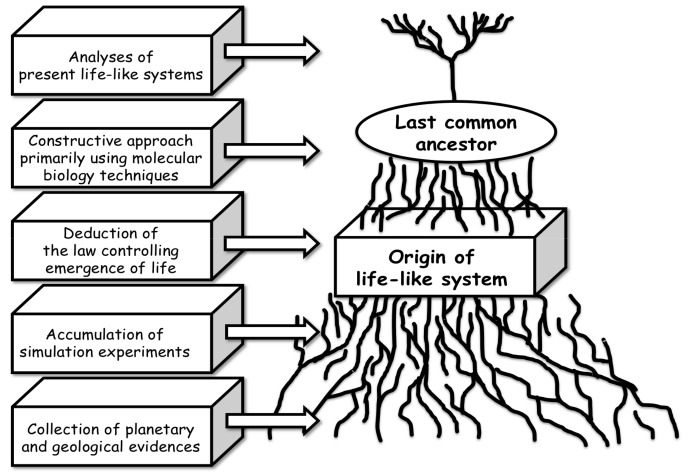
Five approaches to origin-of-life studies.

Scientific approaches to origin-of-life studies may be classified into five different categories (see Figure 1) if origin-of-life processes progressed through chemical evolution on ancient Earth. The first approach is the accumulation of simulation experiments under plausible primitive Earth conditions. This approach provides a number of possible pathways and conditions for the formation of biologically important molecules, such as genetic material [1,2,3,4,5,6,7,8,9,10,11,12,13], amino acids, and protein-like molecules [14,15,16,17,18,19,20,21,22,23,24], in order to construct a primitive life-like system. Although the definition of life and the meaning of a life-like system are important, these are not the main goals of this review. All that can be stated is that a life-like system would be a system located somewhere between chemical networks and cell-type organisms. Detailed discussions of this can be found in my previous publications [25,26]. This approach would clarify what type of chemicals would have formed in the simulated, most plausible, environments. Simulation experiments also involve attempts to construct life-like systems in laboratories, such as in vitro selection of functional RNA [27,28,29,30,31,32,33,34] and artificial cells [35,36]. These simulation experimental data enable a scenario about the origin of life to be drawn up accurately.

The second approach is known as a constructive approach, which attempts to construct life-like systems from simple elements, mostly based on molecular biological technologies. In recent decades, an approach based on the observation of present organisms has developed rapidly because of the success of molecular biology. Specifically, achievements in molecular biology led to the construction and evaluation of the RNA world hypothesis. A selection of artificial functional RNA molecules [28,29] and peptides [37] can be created in vitro to determine whether these RNA molecules could form a life-like system. Recently, several types of evolutionary systems have been developed [38]. However, this approach is limited because the experiments can only be carried out under extremely controlled conditions using pure materials. Of course, molecular biological techniques and instruments were not present on primitive Earth. At the same time, knowledge should be consistent with the geological information about primitive Earth.

The third approach is collecting accurate information of primitive Earth environments. Physicochemical factors include temperature, pressure, pH (for an aqueous phase), minerals, and wet-dry conditions. Recent geological and planetary investigations are improving knowledge of the period between the formation of the solar system and the oldest evidence of life on Earth environments. For instance, a theoretical model for the formation of the solar system implies a very early history of Earth environments [39]; the detection of zircon helped to deduce that the ocean would have been present in around 4.4 Gya [40,41] as well as identifying the age of the post-magma-ocean [42]; and evidence of the realistic age of late heavy bombardment would affect the scenario of chemical evolution leading to the formation of the most primitive life-like systems [43].

The fourth approach is to trace present life-like systems back to relatively primitive organisms, such as prokaryotes and related systems, including viruses and viroids. This aims to extract the essential characteristics of the most primitive life-like system. For instance, estimation of the last universal common ancestor of present organisms [44,45,46,47,48,49] and the origin of genetic information [50,51,52] have been extensively carried out. These four approaches towards the origin of life, from two different directions, might be categorized as bottom-up and top-down approaches, where simple prebiotic reactions and present life-like systems would merge into a simple life-like system. 

The fifth approach is to identify a general law for the emergence of life-like systems, which could be derived from observations of chemical evolution reactions and investigations of present life-like systems. Chemical evolution involves multiple chemical pathways that form different precursors for the emergence of life, some of which are related to the emergence of life and some of which are not. Molecules should have interacted chaotically to form chemical networks, resulting in the core of a life-like system. Finally, the network would have constructed a single system, which behaves as a life-like system. If the laws hidden in these processes were to be identified, the emergence of life on primitive Earth could be understood; for example, the law that dominates self-organization from such complicated and chaotic chemical networks [26,51,52,53]. 

**Figure 2:**
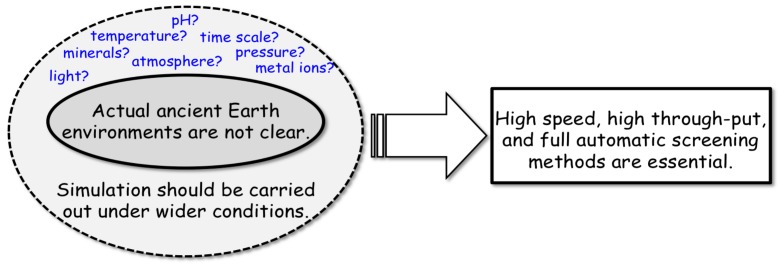
Difficulties of chemical evolution simulation experiments.

Regarding the different approaches, the general difficulty of origin-of-life studies can be summarized as follows (Figure 2). The actual environment between the start of chemical evolution and the formation of the most primitive life-like system is not clear. Estimating the environments would involve many possibilities, depending on the age and location on Earth. For instance, neither the time period [54] nor the temperature when life originated [55,56,57,58] is identified. Naturally, knowledge about the environments on primitive Earth has been and will be improved based on improvements in Earth science. However, it is difficult today to determine whether chemical evolution might have occurred under specific or universal conditions on primitive Earth. 

A strategy that makes an abundant number of simulation experiments necessary is also related to the question of how we evaluate experimental data. For instance, the simulated environments of Miller’s experiment are no longer readily accepted as primitive Earth environments; although the importance of the experiment for origin-of-life studies is generally appreciated, the importance of Miller’s experiment is unwavering. The knowledge about primitive Earth’s atmosphere became more accurate than that at the time of Miller’s experiment. Comparison of Miller’s experiment and similar simulation experiments later indicate that different chemical evolutions also proceed under different environments, even though simulation experiments regarding the origin of life should be carried out more extensively by focusing on plausible environments. 

On the other hand, the possible environments are frequently considered to have been extreme, including high pressure, high temperature, and highly acidic or alkaline conditions. By contrast, the chemical environments within modern organisms are regarded as fairly mild. Thus, the traditional experimental approaches of biochemistry and molecular biology are not useful for such simulation experiments with extreme conditions. Because of this situation, the development of experimental techniques for chemical evolution is essential for facilitating origin-of-life studies. Although such inventions have been important for a long time, they are insufficiently developed. Consequently, the first objective of scientists in this field, including our group, is to focus on the development of research tools for chemical evolution.

## 2. Development of Hydrothermal Microflow Reactor Systems

### 2.1. Importance of Hydrothermal Systems in Relation to the RNA World Hypothesis

Recent investigations on the function of RNA molecules have elucidated that they possess a great diversity of biochemical functions beyond ribozymes and traditional mRNA, tRNA, and rRNA. RNA molecules are the second largest component in cells on a weight-basis [59,60]. Formerly, it was believed that RNA molecules only maintain and transform genetic information during protein-processing directed by DNA sequences. Discovery of the ribozyme suggested that RNA molecules played central roles in the emergence of life-like systems under primitive Earth conditions, because RNA molecules preserve both the genetic information and the enzymatic functions of primitive life-like systems. This is termed the RNA world hypothesis [27]. A few researchers prior to the elaboration of the RNA world hypothesis focused on answering how RNA or RNA-like molecules evolved under primitive Earth conditions. Orgel and co-workers pointed out the importance of the prebiotic formation of RNA molecules under primitive Earth conditions, which anticipated RNA machinery in the 1960s [61]. Successful studies have clarified the question of how RNA and related molecules accumulate under primitive Earth conditions. Moreover, Eigen proposed an independent hypothesis about the origin of genetic coding and information, known as the hypercycle theory, in the early 1970s [50]. There are different studies that support the RNA world hypothesis, but they are not described in this article. 

**Figure 3:**
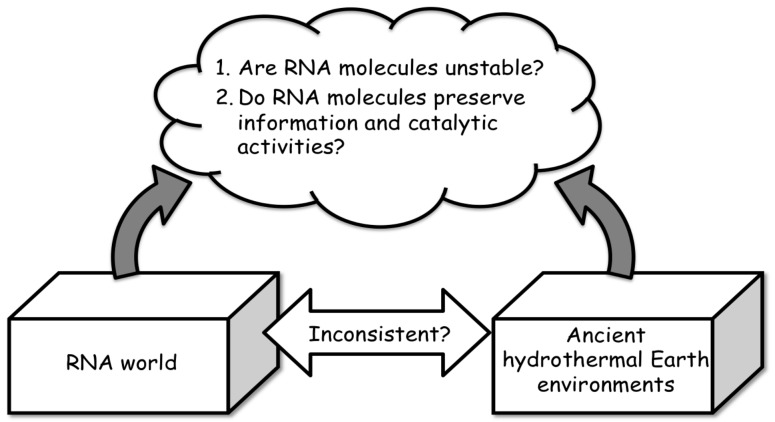
Inconsistency between the RNA world and ancient earth environments.

Biochemists and molecular biologists seem to believe that RNA molecules are not stable. At the same time, it is considered that this argument is ambiguous and not quantitative [62]. Thus, one may assume that the RNA world hypothesis is incompatible with primitive Earth environments (Figure 3). This impression would have resulted in the assumption that RNA molecules are not resistant to extreme environments. Such perceptions are probably due to experiences regarding the influence of ribonucleases, which molecular biologists frequently struggle with. Nevertheless, according to our experience with simulation experiments of prebiotic RNA formation [12,63,64,65], where RNA molecules are normally much shorter than those treated in molecular biology, we demonstrate that RNA molecules are not really unstable. In addition, we demonstrate that very short and cyclized RNA molecules are more resistant to digestion by regular ribonucleases [8]. In general, long RNA molecules possess a higher probability of cleavage on a number of phosphodiester bonds by ribonucleases. 

Present environments for organisms are generally mild compared to the ancient environments of Earth. In general, for a long time it was not believed that a strong relationship between hydrothermal systems and biochemistry exists (Figure 3). However, knowledge about thermophilic organisms has been accumulated in recent decades [44,66,67,68]. Around the hydrothermal vent under the deep ocean, we can find different thermophilic organisms, not only bacteria, but also higher forms of life, living under extreme conditions and without solar energy. Nowadays, it is known that some organisms survive and grow at temperatures over 100 °C. In addition, the presence of diverse microbial communities in relation to hydrothermal fluids has been observed in the oldest life records [69]. Within these organisms, biomolecules are capable of maintaining their biochemical functions under such extreme conditions; however, we do not know the detailed mechanisms that permit these functions, such as genetic and enzymatic functions, to be maintained at temperatures over 100 °C and up to 120 °C. Even RNA molecules can preserve their functions within such thermophilic organisms; therefore, this fact indicates that RNA molecules are stable, at least within the time scale where thermophilic organisms survive. Naturally, the stability of biomolecules in relation to the RNA world hypothesis has been measured in earlier studies [68,70,71,72]; the simple life-time of biomolecules is not suitable as an indicator, since the accumulation of biomolecules is primarily determined by both the formation rate and the degradation rate [25,62]. Thus, comparison of the stability of biomolecules with geological time scale is not sufficient for estimating the accumulation behaviors of biomolecules under hydrothermal environments.

The temperature of primitive Earth should have been high [55,56,57,58], although its history is not yet quantitatively identified. In addition, some extreme natural phenomena, such as volcanic activity and late heavy bombardment, should have increased the temperature of the Earth’s surface [43]. Presumably, the temperature decreased gradually, resulting in the formation of the ocean under a certain atmospheric pressure. The age when a life-like system originated and the temperature at the time of emergence of life are not yet clear. At the same time, it is true that liquid water was necessary for the formation of primitive life-like systems. Continuous research on the phylogenetic tree of life suggested that the nature of the last universal common ancestor was a hyperthermophilic organism, although this argument is still being discussed [45,46,47,48,49]. Possibly, some unknown organisms, which could survive much higher temperatures, might be discovered in the future wherever liquid water is present [68,73]. Thus, the possibility that life emerged under high temperatures, where water existed as liquid, should not be discarded. Recently, hydrothermal environments have also been expected to serve as a possible location for chemical evolution elsewhere in the solar system, such as Europa and Enceladus [74,75]. Hydrothermal conditions are important from this astrobiological viewpoint.

The presence of liquid water is determined by both pressure and temperature; furthermore, the presence of liquid water is possible up to the critical point of water (374 °C, 22.0 MPa). Thus, simulation experiments under a wide range of temperatures should be applied to evaluate what range is suitable for chemical evolution [76,77,78,79,80]. High-temperature conditions possess both advantages and disadvantages for the chemical evolution of biologically important molecules. At these high temperatures, reaction rates increase with increasing temperature, but degradation rates increase as well. On the other hand, the selectivity of main reactions against side reactions should be reduced with increasing temperature, because the activation energy of chemical reactions regarding biopolymers generally decreases with temperature [63,64,65,81]. 

### 2.2. Hydrothermal Flow System

**Figure 4:**
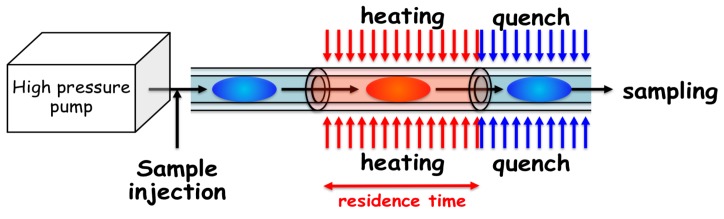
Principle of the hydrothermal microflow reactor system consisting of narrow tubing.

Given this background, we started investigations to solve the apparent paradox between the RNA world hypothesis and the hydrothermal origin-of-life hypothesis. Initially, we attempted to measure the degradation of nucleotides and related molecules in an aqueous solution at high temperatures using conventional batch reactors [71,82]. At once, we realized that the degradation of nucleotides occurs rapidly at temperatures over 150 °C (using traditional methods); therefore, sample analysis was not accurately carried out. The heating time of a sample solution up to a target temperature (heat-up time) is too long to adjust accurately using traditional high-temperature vessels. Thus, we started developing a real-time monitoring system using a microflow reactor, where the heat-up time will be successfully reduced to a sub-millisecond time scale [83]. In the present review, the technical achievements, including the applications and future potential of microflow reactor systems, are summarized based on our previous publications. Using this system, the temperature and the residence time that the sample is exposed to high temperatures can be readily controlled. First, we successfully reduced the residence time to the millisecond time scale [63,84]. The principle of the hydrothermal flow system is illustrated in Figure 4. The goal of the system is for the residence time and heat-up time to be accurately controlled to a millisecond time scale at very high temperatures, possibly over the critical point of water, rather than running simulations of a realistic hydrothermal vent system, in which the circulation time scale through the vent systems frequently reaches several years. The minimum residence time was successfully reduced to 2 milliseconds. The upper temperature limit should be the temperature of natural hydrothermal vent systems in the deep ocean, where the maximum temperature reached 400 °C, and the residence time corresponds to the reaction time scale of biochemical reactions in modern organisms. Thus, simulation experiments at a wide range of temperatures should be applied to evaluate what range is suitable for chemical evolution. The sample solution can be readily withdrawn downstream of the flow system and then analyzed using several analytical methods, such as high-performance liquid chromatography (HPLC). 

### 2.3. Details of the Mechanical Characteristics of the Flow System

**Figure 5:**
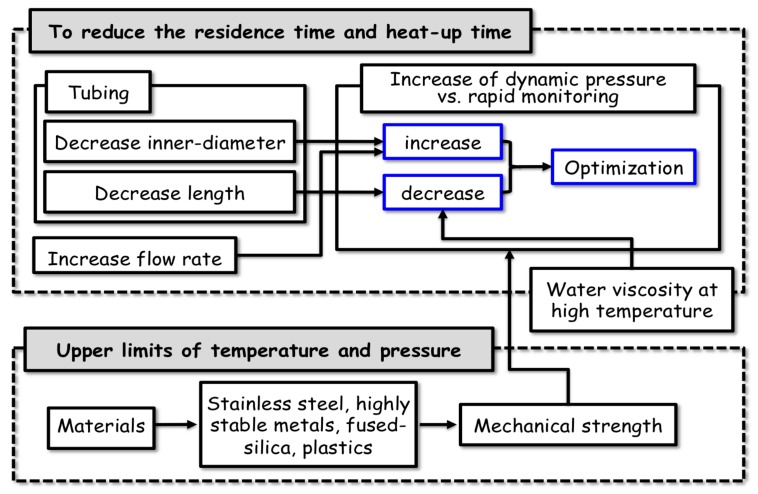
Difficulties faced by the hydrothermal microflow reactor system in the reduction of the residence and heat-up times.

Here, several technical points are briefly described based on our previous publications. Detailed characteristics of the flow system using narrow tubing are summarized from five viewpoints as follows:

(1) Around the time we started to develop the hydrothermal microflow reactor system, there were successful pioneer investigations using flow reactor systems for hydrothermal reactions [85,86,87]. These techniques focused primarily on spectrophotometric approaches to observe more general chemical reactions in aqueous solutions at high temperatures. Several types of high-temperature/pressure resistant cells were designed for hydrothermal reactions using ultraviolet (UV), visible, and Raman spectroscopy for detection. The important point of the present flow system is that it consists of very narrow tubing instead of a small-sized high-temperature/pressure resistant cell. This enables a very short residence time for the sample traveling in the high-temperature reactor, and rapid heating of the sample solution to the target temperature. The strategy and technical difficulties of the flow system are summarized in Figure 5.

After our successful studies using narrow tubing [63,84] for the development of hydrothermal flow reactor systems, hydrothermal flow reactors for the simulation of hydrothermal systems, including the circulation of water in the submarine hydrothermal vent system in the deep ocean, were developed in parallel [19], or modified by some groups [88,89]. 

The advantage of our system is that it is able to control the residence time, covering the millisecond time scale. This enables the analysis of kinetic behavior in real time in a millisecond-to-second time scale. Thus, the limit of the residence time of a sample traveling in high-temperature tubing in the microflow reactor system was evaluated from the viewpoint of hydrodynamics. By decreasing the inner diameter and the length of the tubing, the residence time can be reduced. In addition, if the flow rate of the high-pressure pump and the inner volume (the length and inner diameter of narrow tubing) is changed, the residence time can be controlled. Several parameters have to be balanced in order to reduce the residence time, which complicates this operation: the relationships among the minimum limit of the residence time; the size of tubing; the maximum pressure of the flow system; and the heat-transfer rate [63,84]. The pressure limit of the flow system is naturally determined by the pressure limit of tubing materials, namely the high-pressure pump and connecting devices. Currently, the pressure limit of a commercially available high-pressure pump is 30–50 MPa; additionally, the pressure limits of tubing materials and connecting devices are also adapted. Because the pressure of a natural submarine hydrothermal vent system can be up to 30 MPa, commercially available equipment can be applied in order to construct the present system. Thus, we examined the relationship between pressure and temperature resistance, the heat-transfer rate of different tubing, and the limit size of tubing, by manufacturing different shapes of heating-blocks.

(2) Tubing materials were also inspected to achieve a minimum residence time. Commercially available narrow tubing, including stainless steel (SUS), polytetrafluoroethylene (PTFE), polyetheretherketone (PEEK), and fused-silica capillary (FS) were examined. Naturally, the mechanical strength decreases at high temperatures in the following order: SUS > FS > PEEK and PTFE; and the heat transfer rate decreases in the order SUS > FS > PEEK ~ PTFE. The minimum inner diameter is 0.1 mm for SUS, 0.25 mm for PTFE, 0.13 mm for PEEK, and 0.005 for FS tubing. Consequently, PEEK and PTFE tubing were not suitable for use in extreme high-temperature measurements; PTFE tubing generally broke at temperatures over 180 °C; and PEEK tubing broke at temperatures over 250 °C. Indeed, plastic materials are useful for experiments below upper-limit temperatures due to their ease in handling. SUS tubing did not show any problem in either the heat-transfer rate or the mechanical strength; even though several studies try to use more inert materials. Although the corrosion of SUS surfaces by water at high temperature should be monitored, surface corrosion was not observed in the present system. This is due to a residence time in the millisecond-to-second time scale. Although the upper-limit temperature for FS tubing is commercially stated as 360 °C, it was capable of withstanding temperatures up to 400 °C. Consequently, SUS and FS tubing are primarily suitable for use in the present microflow reactor systems. In addition, 50 MPa is sufficient for keeping the sample solution as a liquid inside the reactor at temperatures up to 400 °C. Both SUS and FS tubing possess sufficient mechanical qualities to resist 50 MPa at high temperatures.

(3) Evaluation of the heat-transfer rate was carried out. Once a sample solution is flowing into the narrow tubing heated at a high temperature, the sample is heated immediately from close to room temperature to up to 400 °C by heat transfer through a heat block. The heat-up time is regarded as an error for the residence time of a sample in the high-temperature narrow tubing. Thus, the heat-up time requires that the residence time be accurately controlled so that a short heat-up time will enable a short residence time. The heat-transfer rate decreases in the following order: SUS > FS > PTFE ~ PEEK [63,84]. SUS and FS have a sufficiently large heat-transfer rate. According to our investigations, a pre-heater is not always necessary in the present system [63]. The relationship between the size and the approximate residence time is summarized in Table 1.

(4) Dynamic pressure in a fluid flow through narrow tubing increases significantly as the inner diameter of the tube becomes narrower. The dynamic pressure that emerges in narrow tubing is inversely proportional to the 2nd power of the inner diameter and is proportional to its length at a constant length-based flow rate [63]. Thus, at a constant volume-based flow rate (e.g. mL/min), the dynamic pressure in the tubing is inversely proportional to the 4th power of the inner diameter. For instance, the dynamic pressure in tubing at a constant flow rate is 16-fold if the tubing’s inner diameter is reduced to half. Thus, to reduce the inner diameter of the tubing, the length of the tubing should be shortened simultaneously.

The dynamic pressure does not give any trouble if tubing with a 0.1–0.25 inner diameter is used. However, the narrower FS tubing gave a strong dynamic pressure, meaning the flow rate needed to be reduced. The actual heat-up time was 2 ms using tubing of 0.025 mm internal diameter and 10 cm (inner volume: 0.04908 μL) at a flow rate of 0.1 mL/min. It is noteworthy that the viscosity of water decreases with increasing temperature (approximately 1/10 from room temperature to 250 °C). This is helpful for handling the flow system by gradually increasing flow rate with rising temperature in order to reach the target temperature. 

(5) Sample broadening within the tubing is also important for accurate measurements. Theoretically, the flow rate is zero at the wall and is fastest at the center of the tubing. The average residence time of a sample at high temperatures can be readily determined by the inner volume (mL) of the heated tubing divided by the flow rate (mL/min) in the following equation, if there is assumed to be no broadening. If the broadening becomes large, the average residence time would not reflect the actual residence time. According to the experiments, the broadening was evaluated as approximately 1.6 times the original sample solution volume [90].

Consequently, the best candidate tubing is normally selected by the residence time scale, that is, FS with the inner diameter < 0.1 mm for 0.002–0.1 s and SUS with inner diameter ≧ 0.1 mm for 0.1–200 s.

According to the principle of a hydrothermal microflow reactor, several technical issues have been investigated for fitting flow reactor systems for actual hydrothermal environments. Moreover, different methods were developed as shown in Table 2. The characteristics of these flow reactor systems are described in the following sections.

### 2.4. Kinetic Measurements by the Flow System

**Figure 6:**
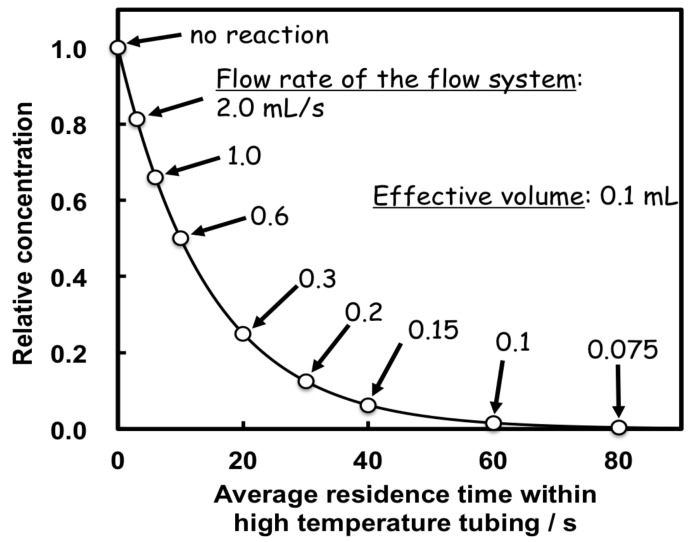
Relation between residence time and flow rate using 0.1 mL tubing.

By changing the flow rate in the flow system, reaction samples with different residence times, exposed at a target high temperature, can be readily collected from the sampling port [63]. The samples can be analyzed through several analytical techniques. For instance, the residence time at a high temperature using 0.1 mL inner volume tubing (0.25 I.D. × 203.7 cm), is controlled for 120 s, 60 s, 30 s, 12 s, 6 s, and 3 s by changing the flow rate to 0.05 mL/min, 0.1 mL/min, 0.2 mL/min, 0.5 mL/min, 1.0 mL/min, and 2.0 mL/min, respectively (Figure 6). By using tubing with an inner diameter of 0.05 mm or narrower, the residence time can be controlled at the millisecond time scale. Consequently, the residence time can be controlled from 2 ms to 200 s in the present microflow reactor system. This is surprising, because it covers an extensive (10^5^) range of the reaction time. This result indicates that we can readily carry out kinetic studies using the present system.

### 2.5. In situ Measurement of Absorption Spectra

Here we describe an application for the principle of hydrothermal microflow reactor systems using narrow tubing for in situ measurement of absorption spectra [92,93,94,97,98]. FS tubing is transparent in the UV, visible, and NIR region. Thus, we realized that in situ observation is possible if a light source is connected to FS tubing using optical devices. 

**Figure 7:**
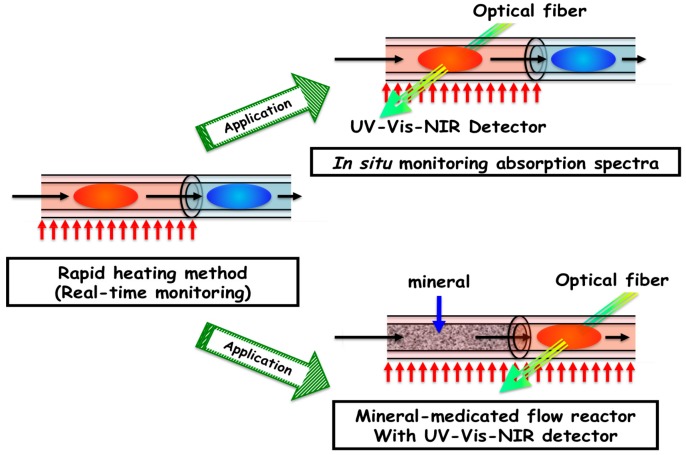
Application of the microflow reactor system to in situ spectrophotometry and the mineral-mediated flow reactor.

The principle of an in situ monitoring system for the reactor is illustrated in Figure 7 (right top). It was surprising that FS tubing was best connected directly with optical fibers to a detection instrument in which no lens system was necessary. This method succeeded and confirmed that in situ observations of absorption spectra at the UV to NIR range are possible. This method is very simple compared to the design of a special spectrophotometric cell to measure absorption spectra [85,86,87]. 

A variety of uses is possible because this system can be regarded as a simple spectrophotometer; this enables measurements of absorption spectra at temperatures up to 400 °C. For instance, absorption spectra deduce reaction behaviors, and equilibrium and kinetic behaviors of chemical species in solutions. In addition, this system possesses an advantage because absorption spectra can be obtained within a shorter time range; therefore, chemical species are not degraded. Based on this advantage, we attempted to measure equilibrium constants at high temperatures [92,94]. Examples will be discussed in a later section.

### 2.6. Mineral-Mediated Hydrothermal System

The principle of the present flow system can be applied to different types of simulation experiments under hydrothermal systems. Applications in more practical simulations regarding hydrothermal and solvothermal systems are also possible, because actual hydrothermal systems are more complicated and diversified [99]. Here, we describe a method used to simulate a heterogeneous system consisting of a high-temperature aqueous phase and a mineral phase [24]. Although the present hydrothermal flow system is useful, it was not simulating realistic hydrothermal vent systems. In actual hydrothermal vent systems, high-temperature water flows through crust minerals on the deep ocean bed. The mineral–aqueous interface is important for the chemical evolution of biopolymers, such as clay minerals [8,10]. Thus, we attempted to simulate these two-phase systems based on the microflow reactor. One difficulty regarding simulations of hydrothermal reactions in the presence of minerals is that the reactions occur too rapidly using conventional techniques. The present flow technique successfully solved this problem. 

The system, which is applied to a mineral-mediated flow reactor, is illustrated in Figure 7 (right bottom). The system consists of almost the same units, except for the mineral-mediated hydrothermal flow reactor, which involves a narrow tube reactor containing mineral particles. Minerals are grained particles (or powders) that are packed inside tubing. Metal tubing is used because of its high-temperature and pressure-resistance qualities. Although different sizes (20–2000 μm) in different mineral species are examined for packaging purposes, some minerals were not used for this system because they swelled, stacked, or were released. It can be noted that the advantageous uses of the mineral-mediated flow reactor, such as kinetic measurements and in situ observations under hydrothermal conditions, are the same as regular microflow reactors. Thus, these particle-packed reactors are not really the same as micrometer-scale structures of chimneys in present black and white smokers, since the hydrothermal phase would mainly interact with the surface of mineral particles rather than with the microstructure of minerals. Nevertheless, the present mineral-mediated hydrothermal flow system is a powerful tool for monitoring reactions under hydrothermal conditions in the presence of minerals. This system applies to hydrothermal systems in the presence not only of naturally occurring particles, but also synthesized particles. 

Recently, the spectrophotometric system and the mineral-mediated flow reactor were combined to generate reactions for the detection of UV-Visible absorption spectra [97,98] (Figure 7, right bottom). 

### 2.7. High-Throughput Modifications

Automatic and high-throughput experimental equipment is helpful for accelerating simulation experiments. Recently, the automation and miniaturization of flow analytical techniques have improved rapidly and, consequently, such technological advances can be applied to the present hydrothermal flow system. In addition, detection devices have also improved. Here we discuss the possible future applications of such techniques. 

We attempted to attach an automatic sample injector to the system so that a very high throughput analysis of samples could be developed [94,95]. Indeed, we demonstrated the flow system for high-throughput analyses of metal ion quantifications, named hydrothermal flow injection analysis. The technique enables a 60-sample analysis within 1 hour. This technique is readily applied to reaction screening regarding chemical evolution under hydrothermal systems in the presence of minerals. 

## 3. What Information Regarding the Origin of Life Can We Obtain from a Hydrothermal System?

### 3.1. Degradation and Formation of Biomolecules

In general, the stability of biomolecules was mostly analyzed using a conventional technique where a sample is sealed in a high-temperature/pressure resistant vessel for heating and the sample is analyzed through general analytical methods [68,70,71,72,100]. As mentioned above, the batch method limits the residence time of samples at high temperature, so the real time measurement within a short time scale at very high temperatures was not possible. Estimation of the stability of these molecules, which are rapidly destroyed, is only possible if the reaction rate measured at lower temperatures is extrapolated to higher temperatures. Naturally, the influence of the side reactions cannot be predicted by simple extrapolation of the data to high temperatures, because the side reactions are not frequently seen at low temperatures. Thus, real-time monitoring within a short time scale is essential for evaluating the stability of biomolecules. 

The stability of biopolymers was extensively determined using the hydrothermal flow system [12,22,63,64,65,81,94]. During these experiments, spontaneous formation of oligopeptides was also discovered [22,23]. The stability of nucleotides, nucleoside, nucleotide bases (250–315 °C [63]), short and long oligonucleotides (150–240 °C [64,65]), amino acids regarding recemization (225–275 °C [81]), and peptides (250–290 °C [22]) has been determined using the hydrothermal flow system (Table 3). The results indicate that biomolecules are degraded at very high temperatures, such as 300 °C, within the millisecond-to-second time scale. In addition, the results demonstrate that DNA is more unstable than RNA at 300 °C, which is somewhat different from the stability of RNA and DNA at low temperatures. The stability of amino acids is ca. 10,000-fold higher than that of nucleotides and the stability of RNA is ca. 100-fold higher than that of peptides. Typically, the degradation of these molecules occurs within the millisecond-to-second time scale at 200–300 °C. According to the results, it can be emphasized that the stability of biomolecules must be evaluated based on the fact that life-like systems are constructed in thermodynamically open systems, where energy sources and materials inflow and outflow [25,26,62,101]. Thus, a straightforward comparison of time scales between the geological time and the stability of these molecules is not meaningful. The accumulation of long biopolymers is an essential step for the emergence of life-like systems, because biopolymers are essential to biochemical functions. Interestingly, the inflow and outflow of biopolymers, in addition to the formation and degradation of biopolymers, determine the accumulation. Furthermore, the stability of biomolecules should be considered based on what type of primitive enzymatic reactions emerged at the beginning of life-like systems. 

### 3.2. Interaction of Biomolecules under Hydrothermal Conditions

In situ absorption spectrophotometers enable equilibrium analyses, which are normally carried out at low temperatures, by using conventional absorption spectrophotometry. Weak interactions such as hydrophobic interactions and hydrogen bonding, as well as electrostatic interactions, are important for the biochemical functions of biomolecules. Thus, three-dimensional structures of biopolymers are mainly constructed on the basis of electrostatic interactions, hydrophobic interactions, and hydrogen bonding in aqueous solutions at low temperatures. However, hydrophobic interactions and hydrogen bonding would be inefficient at very high temperatures because they become weak. We attempted to detect the effect of temperature on these interactions using the present in situ monitoring method. Three-dimensional structures should be effective within thermophilic organisms at high temperatures.

**Figure 8:**
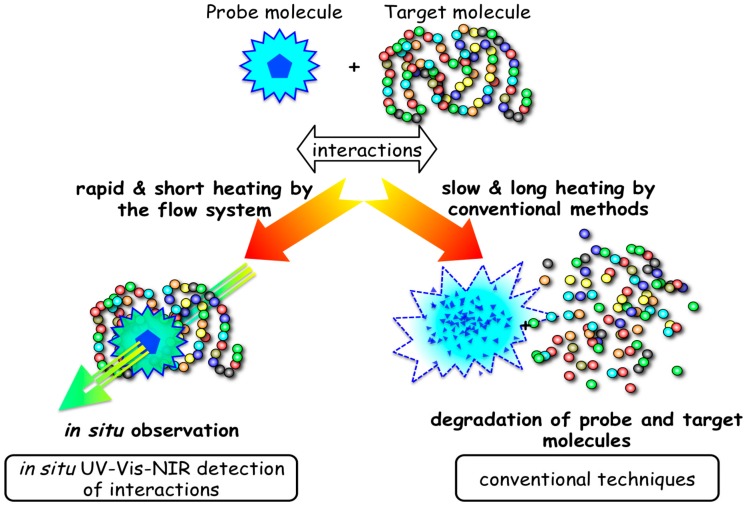
In situ observation of the interaction between probe molecule and a target biopolymer.

The principle of in situ monitoring of molecular interactions is illustrated in Figure 8. Conventional batch reactors with spectrophotometric detectors expose samples to high temperatures for long periods. Thus, both the probe molecules and the target molecules are easily destroyed. By contrast, the present system enables the measurement of absorbance within a time scale where both the probe molecules and the target molecules are not degraded, but interactive. Here, two examples are briefly described. 

First, DNA interactions were observed using ethidium bromide in the presence and absence of MgCl_2_. Double-stranded DNA is normally denatured to single-stranded DNA, which was readily observed at about 75 °C using the present system [93,102]. At much higher temperatures, we observed an increase in the absorption spectra at a wide range; it was confirmed that this was due to turbidity resulting from the formation of insoluble single-stranded DNA at temperatures over 100 °C. At temperatures over 200 °C, the turbidity disappeared, because the DNA was degraded to short oligonucleotides, including monomeric nucleotide, due to the cleavage of phosphodiester bonding. Although the behavior of DNA at temperatures up to around 100 °C can be observed by merely using a conventional spectrophotometer, the behavior of DNA at temperatures much higher than 100 °C is possible by using the present in situ measurement flow system.

Second, the association of proteins with chromogenic agents was successfully measured [94]. We attempted to analyze interactions using normal proteins, which are active at low temperatures. We confirmed that most proteins are readily denatured and precipitate at temperatures below 100 °C, and then found that bovine serum albumin (BSA) did not denature at over 100 °C, where it was possible to measure its association with the chromogenic agent. BSA gradually, not instantly, denatures at temperature over 125 °C. At the same time, the stability of chromogenic agents was also examined and a water-soluble porphyrin (TPPS) was chosen due to its high-temperature resistance. In general, the association between such proteins and chromogenic agents proceeds within a very short time range because the reaction occurs in a diffusion-controlled step. Results support the notion that TPPS simply attaches to BSA. Consequently, we were able to monitor the association between BSA and TPPS because the heat-up time is much shorter than the degradation of these molecules. The present system is readily applicable for several equilibrium analyses where degradation or denaturing does not occur within a millisecond time scale. 

### 3.3. Chemical Evolution of Biomolecules under Hydrothermal Conditions

Based on these data, we evaluated the possibility of emergence of life-like systems under hydrothermal conditions. First, we cannot determine that the hydrothermal origin-of-life hypothesis and the RNA world hypothesis would be inconsistent on the basis of the principle that the accumulation of biomolecules is determined by both the formation rate and deformation rate, as well as the inflow rate and outflow rate of the system [25,26,62,101]. Second, these studies implied a possible upper limit of temperature for the emergence of life [101,103,104]. Third, these results suggested the importance of considering both the formation and deformation of biomolecules during chemical evolution. Biomolecules, such as proteins and nucleic acids, are not stable under hydrothermal conditions as per our perception and geological time scale. However, if formation and degradation is controlled within a very short time scale, the accumulation of these molecules could occur under such extreme conditions. Fourth, these data successfully demonstrated the importance of the interactions among biomolecules and the solubility of biomolecules under hydrothermal environments, even though these are not the focus of most scientists in this field.

### 3.4. Strategy of Simulation Experiments for Chemical Evolution towards the Origin of Life

We almost succeeded in establishing our earlier goal for the development of hydrothermal microflow reactors, which are powerful tools for observing stability and chemical evolution directly under hydrothermal conditions [12,24,63,90,91]. At the same time, technical problems regarding the origin-of-life study were highlighted by these studies.

Our hydrothermal flow systems can be applied in different types of simulated hydrothermal conditions, with and without minerals, and for measurements of in situ absorption spectra in the UV, visible, and NIR regions. Hopefully, this may be applied to chemical evolution processes, which are not yet clarified, such as the emergence of chirality [81]. However, there is still potential for further improvements of the system. For instance, although the mineral-mediated hydrothermal flow reactor can be applied for investigating the roles of minerals in chemical evolution, some minerals, such as clay minerals, could not be inspected due to clogging of the flow reactor. In addition, other different types of spectroscopy could be attached with the flow systems. The applications could be focused on in situ florescence spectroscopy, Raman scattering spectroscopy, CD spectroscopy, neutron scattering, among others.

**Figure 9:**
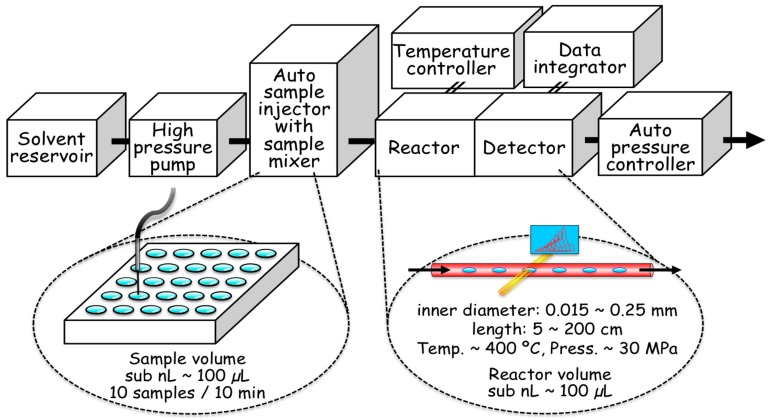
Full automation of microflow reactor system with in situ spectrophotometric detector.

At the same time, the plausible environments on primitive Earth remain ambiguous and might not be clearly understood in the foreseeable future. A possible approach is to run a number of routine experiments to accelerate screenings by automation. This would also be advantageous because possible environments of chemical evolution could be covered, some of which might be present on other planets. Here, it can be pointed out that recent automation techniques could be applied to this field. Fortunately, the present system belongs to the category of flow analytical techniques, so that several modern techniques including computer automation can be readily applied [105]. Naturally, an automatic sample injector, a full automatic pressure controller, and other components can be connected to the system. A small sample size, 10–100 μL, is also useful as a background for full automation of such screening experiments [95,96]. Actually, the development of the present method accelerates the acquisition of experimental results. For instance, a single reaction curve including more than 10 data points can be carried out at least within one workday, and will be analyzed by HPLC within one day. Thus, chemical reactions regarding the origin of life, such as the degradation of biopolymers, can be readily analyzed at very high temperatures. A possible set-up for a full automatic instrument to screen chemical evolution reactions with an automatic sample injector and data integrator is illustrated in Figure 9. 

## 4. Conclusions

The present paper has described briefly the history and background leading up to the development of hydrothermal microflow reactor systems useful for origin-of-life studies. Our hydrothermal microflow reactor system resolved the challenge in studies on hydrothermal reactions, and demonstrated that the systems are useful and accurate for investigating the stability and formation of biomolecules in relation to chemical evolution at very high temperatures. Kinetic and thermodynamic analyses are also possible using the hydrothermal flow system; then, interactions of biopolymers and the importance of biomolecules were visualized. The methods covered in this review diversified hydrothermal environments for simulation experiments compatible with Hadean extreme environments deduced from planetary and geological investigations. This paper demonstrates that the present flow techniques are powerful research tools for screening chemical evolution reactions. This viewpoint deduces that the accurate, convenient, high-throughput instrumentation achieved by combining analytical automation and microflow techniques is an important strategy for origin-of-life studies in the near future.

## Figures and Tables

**Table 1:** Heat-up time for different sizes of tubing in terms of length and inner diameter.

Tubing Size		Heat-Up Time (ms)
Length (cm)	Inner Diameter (mm)	
200	0.25	3000
50	0.10	300
20	0.05	40
10	0.025	4
5	0.015	2

**Table 2:** Improvement of hydrothermal microflow reactor systems.

Type of Flow Reactor	Improvement	References
Real-time monitoring of hydrothermal reactions using narrow tubing	Monitoring at 0.002–200 s, at 400 °C, at 30 MPa.	[63,83,84]
In situ monitoring of hydrothermal reactions with UV-visible absorption spectrophotometer	In situ measurement of UV-visible-NIR absorption spectra at 200–600 nm, at 0.3–60 s, at 400 °C, at 30 MPa	[90,91,92,93,94]
Mineral-mediated hydrothermal flow reactor	High-temperature reactor column packed with mineral particles at 0.3–60 s, at 300 °C, 30 MPa.	[24]
Flow-injection analysis for high temperatures with hydrothermal flow reactor	Reactions are accelerated with high-temperature reactor at temperatures up to 400 °C	[95,96]
In situ monitoring in the presence of solid-state catalysts with UV-visible-NIR spectrophotometer	High-temperature reactor column packed with solid-state catalysts and in situ analysis at UV-visible-NIR region.	[97,98]

**Table 3:** Half-life of biomolecules under hydrothermal conditions.

Molecules	Half-Life (s)		
	100 °C	200 °C	300 °C
oligoRNA	2400~4500	2.0~3.4	0.02~0.04
C^3’^pG	13000	29	0.54
dCpdG	570000	46	0.098
Half-Life (C^3’^pG)/Half-Life (dCpdG)	0.023	0.630	5.5
5’-ATP	1300	0.37	0.0019
Alanine	16000000	3400	14
Half-Life (ATP)/Half-Life (Alanine)	8.1 × 10^−5^	1.1 × 10^−4^	1.4 × 10^−4^

The magnitudes of half-life were calculated on the basis of our previous kinetic data shown in references [63,65,71,82].
